# Changes in costs and effects after the implementation of disease management programs in the Netherlands: variability and determinants

**DOI:** 10.1186/1478-7547-12-17

**Published:** 2014-07-28

**Authors:** Apostolos Tsiachristas, Jane Murray Cramm, Anna P Nieboer, Maureen PMH Rutten-van Mölken

**Affiliations:** 1 Institute for Medical Technology Assessment, Erasmus University Rotterdam, P.O. Box 1738, Rotterdam, 3000 DR, The Netherlands; 2 Department of Health Policy and Management, Erasmus University Rotterdam, P.O. Box 1738, Rotterdam, 3000 DR, The Netherlands

**Keywords:** Costs, Effectiveness, Coordinated care, Cardiovascular disease, Diabetes, COPD

## Abstract

**Objectives:**

The aim of the study was to investigate the changes in costs and outcomes after the implementation of various disease management programs (DMPs), to identify their potential determinants, and to compare the costs and outcomes of different DMPs.

**Methods:**

We investigated the 1-year changes in costs and effects of 1,322 patients in 16 DMPs for cardiovascular risk (CVR), chronic obstructive pulmonary disease (COPD), and diabetes mellitus (DMII) in the Netherlands. We also explored the within-DMP predictors of these changes. Finally, a cost-utility analysis was performed from the healthcare and societal perspective comparing the most and the least effective DMP within each disease category.

**Results:**

This study showed wide variation in development and implementation costs between DMPs (range:€16;€1,709) and highlighted the importance of economies of scale. Changes in health care utilization costs were not statistically significant. DMPs were associated with improvements in integration of CVR care (0.10 PACIC units), physical activity (+0.34 week-days) and smoking cessation (8% less smokers) in all diseases. Since an increase in physical activity and in self-efficacy were predictive of an improvement in quality-of-life, DMPs that aim to improve these are more likely to be effective. When comparing the most with the least effective DMP in a disease category, the vast majority of bootstrap replications (range:73%;97) pointed to cost savings, except for COPD (21%). QALY gains were small (range:0.003;+0.013) and surrounded by great uncertainty.

**Conclusions:**

After one year we have found indications of improvements in level of integrated care for CVR patients and lifestyle indicators for all diseases, but in none of the diseases we have found indications of cost savings due to DMPs. However, it is likely that it takes more time before the improvements in care lead to reductions in complications and hospitalizations.

## Background

Chronic diseases pose an increasing threat to population health, enlarge the burden of care giving, and constrain the financial viability of health care systems worldwide. Because these health care systems originate largely from an era where acute and infectious diseases were more prominent, their design is not optimal for chronic care [1]. This triggered many new approaches for providing continuous, integrated, pro-active and patient-centred care by a multidisciplinary team of care providers in order to improve health outcomes and reduce costs. There is evidence that these approaches improve the quality of the care as measured by process indicators like coordination of care, communication between caregivers, patient satisfaction, provider adherence to guidelines, and patient adherence to treatment recommendations [2]. However, there is debate about the impact on health outcomes and efficiency improvements, a debate complicated by large differences in study designs, outcome metrics and target populations across studies [3] as well as cultural and political barriers to evaluation [4].

In the Netherlands, a recently established regulation introduced a bundled payment system to promote disease management programs (DMPs) for patients with diabetes mellitus type two (DMII), chronic obstructive pulmonary disorder (COPD) or at risk for a cardiovascular disease (CVD) event [5]. Although, the wide-scale implementation of DMII-DMPs was smooth and successful, the uptake of DMPs for COPD and cardiovascular risk (CVR) is still troublesome. This is because health insurers, which contract DMPs from care groups, are yet to be convinced about the financial attractiveness of these programs [6]. Illustrative of this scepticism is that the largest Dutch health insurer does not contract CVR-DMPs and provides only a yearly add-on payment per patient with an elevated CVR to cover costs of coordination, provider training and additional ICT support. Another large health insurer contracts CVR-DMPs only for patients diagnosed with a CVD (secondary prevention) and not for individuals at risk to have CVD (primary prevention). In addition, the debate embeds the adequacy of the current single-disease DMPs for patients with multiple morbidities, which seems to be the norm rather than the exception [7].

Therefore, the provision of evidence about the variability in costs and effects of different implemented DMPs is eminent for the successful implementation of integrated chronic care in the Netherlands. This study aims to investigate the changes in costs and outcomes after the implementation of DMPs, to identify potential determinants of them, and to compare the costs and outcomes of different DMPs.

## Methods

### Design and setting

In a prospective pre-post study, we compared 16 different DMPs spread across different regions of the Netherlands [8]: 9 CVR-, 4 COPD-, and 3 DMII-DMPs. Two CVR-DMPs included patients that were at risk for developing CVD (primary prevention), two CVR-DMPs patients that had already been diagnosed with CVD (secondary prevention), and five CVR-DMPs included both patient groups. The implementation of the DMPs and their participation in the evaluation study was financially supported by the Netherlands Organization for Health Research and Development (ZonMw, project number 300030201). Outcomes and health care resource utilization were measured twice, once at the start of the DMP and once after approximately 12 months, using a patient-questionnaire. A detailed description of the design and setting is presented in Lemmens et al. [8].

### Intervention

To describe the details of each DMP we read program documents and interviewed DMP managers using a check-list of possible interventions that may be included in such programs, grouped by the components of the chronic care model [9]. Although the services included in the integrated care package differed between the DMPs, most programs focused on improving the collaboration between different disciplines of health care professionals and redesigning the care-giving process to patient centred care more proactively. Most of them provided interventions such as self-management education and training directed at life-style improvement (physical reactivation, smoking cessation, diet improvement), decision support to implement guidelines and protocols, integration of ICT systems, training for health care providers, case management, and reallocation of tasks between care providers [8,10]. A detailed presentation of the interventions provided by each DMP is provided by Additional file 1.

### Outcomes

We investigated the impact of the DMPs on a broad range of outcomes including changes in care delivery process, patient life-style and self-management behaviour, and health-related quality of life (HR-QoL) [9]. More specifically, we investigated the impact of DMPs on: a) the level of chronic care integration using the Patient Assessment Chronic Illness (PACIC) questionnaire [11], b) patient life-style measured by self-reported smoking status (current, former or never smoker) and physical activity (expressed in the number of days per week that an individual had more than 30 minutes physical activity), c) self-efficacy using the respective subscale of the Self-Management Ability Scale- Shorter (SMAS-S) [12], and d) the 3-level EQ-5D utility scores which were based on the Dutch value set and used to estimate quality adjusted life years (QALYs) [13]. The questionnaire designed to measure these outcomes also included questions about socio-demographic patient characteristics and a checklist of morbidities.

### Costs

We estimated five categories of costs, i.e. 1) the development costs, 2) the implementation costs, 3) the costs of health care utilization, 4) the costs borne by patient for travelling to receive care and 5) the costs of productivity loss due to absence from paid work. When calculating costs from a healthcare perspective cost categories 1, 2, and 3 were included; categories 4 and 5 were added when adopting the societal perspective.

The development costs included all costs made during the preparation phase of DMPs e.g. labour costs for brainstorming sessions, training costs, and ICT support costs. The implementation costs were costs that occurred after the provision of DMP interventions to patients had started and included the costs for managing the DMP, the costs of multidisciplinary team meetings, the costs associated with collecting quality of care indicators for audit and feedback, the costs of materials used for patient education, and the costs of keeping the ICT operating. The development and implementation costs were systematically collected using a template based on the CostIt instrument of the World Health Organisation (WHO) [14]. This template was completed during face-to-face interviews with DMPs managers. During these interviews managers were also asked about the presence of additional funding to cover the specific elements of integrated care. Capital costs were amortized over their life span and allocated to the DMP based on square meters for the costs of buildings, full-time equivalents for the costs of ICT and medical technologies (e.g. spirometer). The sum of the capital costs and the operating costs of a DMP was then divided by the number of DMP participants. The costs of developing a DMP were amortized in 5 years assuming this period as the life span of a DMP since after this period changes in guidelines and governmental policies would probably affect the initial form of a DMP. The development and implementation costs per patient were consequently calculated by adding one fifth of the development costs to the annual implementation costs and dividing it by the number of DMP participants.

The costs of health care utilization were based on a questionnaire asking patients about the number of caregiver contacts (GP, nurse practitioner, nurse, dietician, physiotherapist, podiatrist, lifestyle coach, medical specialists in outpatient clinics etc.), hospital admissions and admission days, and medication use. The recall period for these questions was 3 months and we asked for all health care utilization, whether or not it was related to the disease targeted in the DMP. In addition to these costs, the travel costs of patients were calculated, using their self-reported distance to a health care provider. Finally, the costs of productivity loss due to illness were calculated, using the friction cost approach [15], based on questions about absence from paid employment due to illness. Standard unit costs as reported by [16] were applied. All costs were inflated to 2012 and reported on an annual basis per patient (see Additional file 2).

### Statistical analysis to estimate changes within DMPs

We started with paired Wilcoxon tests and McNemar chi-square tests to investigate whether the differences in costs and effects between the baseline and follow-up measurements were statistically significant. In addition, a multi-level analysis was performed to explore the determinants of change in costs and EQ-5D utilities of patients clustered in DMPs. Generalized linear mixed models were used to accommodate the skewness in the health care utilization cost and EQ-5D data as well as to include predictor variables on patient and DMP level. Predictor variables on patient level included: the EQ-5D or costs at baseline (depending which of the two was the outcome variable), age, physical activity at baseline and its change, the PACIC score at baseline and its change, the SMAS-self-efficacy score at baseline and its change, smoking cessation during the follow-up period, and presence of multi-morbidity. Gender, socio-economic status, and marital status were not included in the final model after performing likelihood ratio tests. Predictor variables on the DMP level included the DMP target population and the existence of additional payments to cover overhead and management expenses provided on top of the usual payment per patient.

To explore the variance in the change in outcomes and costs between DMPs that targeted patients at risk for a first (primary prevention), or subsequent CVD event (secondary prevention), or both types of CVR prevention, we also estimated separate models for these sub-groups.

### Statistical analysis to estimate differences between DMPs

In each disease category, we identified the DMP that was most effective and least effective in improving the patients’ generic health-related quality of life as measured in QALYs. In this manner we identified 5 pairs of DMPs (i.e. for primary CVR prevention, secondary CVR prevention, both types of CVR prevention, COPD, and DMII). For each of the 5 pairs, we calculated the cost-utility of the most effective versus the least effective DMP in terms of incremental costs per QALY gained. These calculations were performed from two perspectives, i.e. the health care perspective (cost category one to three) and the societal perspective (all five categories of costs).

We used inverse probability weighting to balance the two comparators in each pair with respect to age, gender, education, presence of multi-morbidity, marital status, and EQ-5D at baseline. Inverse probability weighting was chosen because it is the preferred propensity score matching technique for small samples [17]. We performed bootstrapping to generate 5,000 samples from the original sample. For each bootstrapped sample we estimated a generalized linear model for each outcome variable (i.e. QALYs or costs) using the inverse probability weights to get the coefficients adjusted for the propensity score of each observation as well as age, gender, education level, multi-morbidity, and marital status. We used inverse Gaussian distribution and power minus two link for the QALY estimation and gamma distribution and log link for the costs estimation. In this manner, 5,000 predicted incremental costs and 5,000 predicted incremental QALYs were generated. Each of the 5,000 ICERs was calculated as the mean of the predicted incremental costs divided by the mean of the incremental QALYs. These predicted ICERs were then plotted on a cost-effectiveness (CE) plane to show the uncertainty in the ICER.

### Sensitivity analysis

The CUA was also performed excluding the development and implementation costs in order to investigate how sensitive the estimated ICERs are to these costs.

## Results

### Sample

As Table 1 shows, there were 2,438 respondents at the baseline measurement and 1,974 respondents at the follow-up measurement. One thousand three hundred twenty two individuals responded to both measurements (i.e. had complete data).

**Table 1:** Sample size per disease and measurement moment

**Disease**	**DMPs**	**Baseline**	**Follow-up**	**Baseline & follow-up**
Total	16	2,438	1,974	1,322
CVR	9	1,342	1,125	725
COPD	4	689	596	395
DMII	3	407	253	202

The sample characteristics by disease are presented in Table 2. The mean age of the total sample was 65.1 years and consisted of 47% females, 38% low educated, 38% employed, and 30% singles. The mean multi-morbidity among the respondents measured by the Charlson co-morbidity index [18] was 1.83. The COPD sample included proportionally more low-educated, unemployed, and single patients than the other two samples. COPD patients were also older and had higher Charlson co-morbidity scores.

**Table 2:** Sample characteristics by disease at baseline

	**CVR**	**COPD**	**DMII**	**Total sample**
	**Mean (sd)**	**Mean (sd)**	**Mean (sd)**	**Mean (sd)**
	**[DMP range]**	**[DMP range]**	**[DMP range]**	**[DMP range]**
Age	64.1 (9.7)	66.5 (10.0)	66.2 (9.7)	65.1** (9.9)
	[59.6;67.8]	[65.4;69.3]	[64.2;67.1]	[59.6;69.3]
% Females	48	48	43	47
Charlson comorbidity index	1.48 (1.10)	2.26 (1.28)	2.22 (0.99)	1.83** (1.20) [1.15;2.48]
% Low education	35	48	25	37**
% Employment	43	30	37	38**
% Single	26	36	30	30**

The table presents the mean (sd) unless otherwise indicated; in [] is given the range between DMPs i.e. lowest and highest values across DMPs in the same disease area; low education was defined as no or only primary education; The p-values show whether the values are statistically different between the diseases **Statistically different at p < 0.01 between the diseases.

Table 3 presents the baseline values of the outcome measures and their change after one year. The perceived level of chronic care integration was the highest at baseline among patients in DMII-DMPs (3.29) and the lowest in CVR-DMPs (2.80). Individuals in CVR-DMPs were the most physically active at baseline (5.00 days per week) while diabetic patients were the least physically active (4.74 days). In addition, the percentage of smokers was the highest in the COPD sample (39%) and the lowest in the CVR sample (21%). Patients in DMII-DMPs had scored the highest in self-efficacy (4.56) and patients in COPD-DMPs the lowest (4.33). The mean EQ-5D utility score at baseline was 0.83 in the CVR sample and 0.84 in the DMII sample while for the COPD sample it was lower (0.79).

**Table 3:** Outcomes by disease at baseline and differences with the outcomes in the follow-up

	**CVR**			**COPD**			**DMII**			**Total sample**	
	**Mean at baseline (sd)**	**Mean change (sd)**	**Range of change across DMPs**^**#**^	**Mean at baseline (sd)**	**Mean change (sd)**	**Range of change across DMPs**^**#**^	**Mean at baseline (sd)**	**Mean change (sd)**	**Range of change across DMPs**^**#**^	**Mean change**	**Range of change across DMPs**^**#**^
PACIC (1; 5 highest = best)	2.80 (0.84)	0.10** (0.80)	+0.02; +0.26	2.92 (0.89)	−0.03 (0.75)	−0.05; +0.06	3.29 (0.85)	−0.23* * (0.72)	−0.27; − 0.18	0.01 (0.78)	−0.27; +0.26
Physically active days per week	5.00 (2.07)	0.33** (2.15)	−0.23; +0.82	4.82 (2.13)	0.37** (2.20)	−0.11; +1.36	4.74 (1.94)	0.29 (2.01)	+0.05; +0.89	0.34** (2.14)	−0.23; +1.36
% smokers	21	−6 pp**	−2.5 pp; −10.7 pp	39	−11 pp**	−7.3 pp;-13.7 pp	22	−9 pp**	−8 pp; −13.6 pp	−8 pp**	−13.7 pp; −2.5 pp
Self-efficacy (1; 6 highest = best)	4.45 (0.87)	−0.28** (0.75)	−0.33; − 0.15	4.33 (0.88)	−0.34** (0.73)	−0.48; −0.27	4.56 (0.85)	−0.29** (0.77)	−0.42; −0.22	−0.30** (0.75)	−0.48; −0.15
EQ-5D (−0.33; 1 highest = best)	0.83 (0.18)	−0.01* (0.16)	−0.06; +0.03	0.79 (0.20)	−0.04** (0.19)	−0.04; − 0.03	0.84 (0.16)	−0.03* (0.14)	−0.04; −0.02	−0.02** (0.17)	−0.06; +0.03

pp = percentage points; *(p < 0.05); **(p < 0.01); the differences are calculated subtracting the outcome values at baseline from the outcome values at follow-up.

### Changes in outcomes

Changes in PACIC scores were significantly positive (0.10) in the CVR sample (range across the 9 CVR DMPs from +0.02 to +0.26) and significantly negative (−0.23) in the DMII sample (range across the 3 DMII-DMPs from −0.27 to −0.18). In the CVR and COPD samples the change in the number of days per week with more than 30 minutes of physical activity was positive and statistically significant (0.33 and 0.37 respectively). The range in physical active days across the CVR and COPD-DMPs was quite large as Table 3 shows. The percentage of smokers decreased substantially in all samples (ranging across all 16 DMPs from −13.7 percentage points to −2.5 percentage points) as well as the self-efficacy (ranging from −0.48 percentage points to 0.15 percentage points) and the HR-QoL (ranging from −0.06 percentage points to +0.03 percentage points).

### Changes in costs

The development and first year’s implementation costs per patient of the 16 DMPs are presented in Table 4. As this table shows, there is large variation in the implementation costs per patient between and within the three diseases ranging from €16 to €1,709. This is due to the variation in the total development and implementation costs and the number of participants per DMP. The largest share of these costs is for costs related to the time that personnel dedicates to the implementation of DMPs. Costs related to educational courses for caregivers and information brochures for patients were low in almost all cases (except in DMII-DMP1). In some DMPs “other” costs such as ICT, energy, and accommodation costs were relatively high (e.g. 66% in DMII-DMP 2).

**Table 4:** Development and implementation costs by DMP

	**N**	Development phase*			Implementation year 1*		
		**Total costs without amortization**^**#**^	**Costs per patient without amortization**	Costs per patient with amortization*	**Total costs without amortization**^**#**^	**Costs per patient without amortization**	**Costs per patient with amortization**
CVR-DMP 1	300	52,136	174	35	16,426	55	90
CVR-DMP 2	207	54,417	263	53	68,415	331	381
CVR-DMP 3	700	98,754	141	28	153,215	219	234
CVR-DMP 4	300	274,783	916	183	171,026	570	605
CVR-DMP 5	550	26,807	49	10	67,604	123	142
CVR-DMP 6	450	27,923	62	12	149,990	333	356
CVR-DMP 7	125	13,324	107	21	37,968	304	387
CVR-DMP 8	250	195,007	780	156	168,385	674	715
CVR-DMP 9	1,000	26,678	27	5	81,258	81	92
COPD-DMP 1	2,508	154,504	62	12	214,239	85	90
COPD-DMP 2	1,600	93,909	59	12	49,751	31	38
COPD-DMP 3	133	49,639	373	75	55,191	415	493
COPD-DMP 4	2,400	44,586	19	4	32,599	14	18
DMII-DMP 1	2,400	5,891	2	0	28,061	12	16
DMII-DMP 2	233	162,889	699	140	387,879	1,655	1,709
DMII-DMP 3	300	50,304	168	34	61,338	204	239

*We used 5 years as amortization period; ^#^These costs are not per patient.

At baseline, patients in COPD-DMPs had the highest mean yearly hospital costs (€1,967), medication costs (€857), total health care costs (€4,368) and total costs (€5,320) while patients in CVR-DMPs had the highest mean yearly productivity loss (€1,648) (see Table 5). Patients in DMII-DMPs had the highest primary care costs (€941). However, almost all differences between baseline and follow-up were statistically insignificant and the standard deviations of the estimated means were large. Only the outpatient costs of patients with diabetes increased by €115. As Table 5 shows, the changes across DMPs within the same disease and between diseases varied largely. The cost change within each disease category ranged from negative to positive across DMPs except for the outpatient costs and inpatient costs of patients with diabetes.

**Table 5:** Costs at baseline and differences with the follow-up measurement

	**CVR**			**COPD**			**DMII**			**Total sample**	
	**Mean at baseline (sd)**	**Mean change (sd)**	**Range of change across DMPs**	**Mean at baseline (sd)**	**Mean change (sd)**	**Range of change across DMPs**	**Mean at baseline (sd)**	**Mean change (sd)**	**Range of change across DMPs**	**Mean change**	**Range of change across DMPs**
Primary care	610 (857)	34 (1,069)	−510; +314	916 (1388)	49 (1,601)	−5; +155	941 (947)	−84 (1,226)	−236; +88	21 (1,273)	−510; +314
Outpatient hospital care	365 (778)	30 (954)	−443; +259	654 (2,488)	−119 (2,524)	−272; +22	338 (604)	115* (809)	+86; +169	−2* (1,583)	−443; +259
Inpatient hospital care^$^	587 (3,526)	624 (9,452)	−551; +2,148	1,967 (13,256)	320 (18,563)	−396; +1,162	701 (3,714)	−454 (4,065)	−1,211; − 220	368 (12,426)	−1,211; +2,148
Medication	370 (362)	3 (261)	−45; +41	857 (601)	3 (417)	−2; +6	518 (482)	1 (318)	−44; +34	3 (323)	−45; +41
Total healthcare utilization costs	1,911 (4,102)	691 (9,812)	−1,107; +2,626	4,368 (14,256)	238 (19,080)	−672; +1,055	2,504 (4,015)	−446 (4,444)	−93; −1,066	382 (12,826)	−1,107; +2,626
Travelling	74 (215)	−2 (344)	−113; +90	226 (1,190)	−109 (1,145)	−328; +47	174 (378)	−22 (441)	−23; −19	−37** (699)	−328; +90
Productivity	1,648 (8,080)	−495 (7,349)	−1,988; +1,075	658 (4,724)	341 (6,603)	0; +459	216 (1,410)	188 (2,656)	−210; +454	−102 (6,571)	−1,988; +1,075
Total costs	3,302 (9,006)	468 (13,559)	−1,893; +4,269	5,320 (15,390)	85 (20,354)	−1,232; +375	3,489 (7,605)	−517 (9,662)	−1,591; − 167	203 (15,448)	−1,893; +4,269

^$^inpatient hospital care costs include also emergency care costs; *(p < 0.05); **(p < 0.01); the differences are calculated subtracting the costs at baseline from the costs at follow-up; primary care costs included contacts with GP, nurse practitioner, nurse, dietician, physiotherapist, podiatrist, lifestyle coach, etc.

In primary and mixed prevention CVR-DMPs, the PACIC was increased by 0.18 and 0.10 and the number of days with at least 30 minutes of physical activity in a week increased by 0.43 and 0.37, respectively (Table 6). The decrease in the percentage of smokers ranged from 3% (primary prevention) to 8% (secondary prevention). As Table 6 shows, self-efficacy was decreased in all three types of CVR prevention by about 0.28 while the EQ-5D decreased in the mixed CVR prevention DMPs by 0.02.

**Table 6:** Costs and outcomes by type of CVR prevention

	**Primary prevention**		**Secondary prevention**		**Mixed**	
	**Baseline**	**Change**	**Baseline**	**Change**	**Baseline**	**Change**
PACIC (1–5 highest)	2.64 (0.77)	0.18* (0.76)	2.52 (0.79)	0.09 (0.75)	2.92 (0.84)	0.10* (0.82)
Physically active days per week	5.25 (1.91)	0.43* (1.94)	5.15 (2.10)	0.12 (2.11)	4.91 (2.10)	0.37** (2.20)
% smokers	13	−3*	30	−8**	20	−6**
Self-efficacy (1–6 highest)	4.44 (0.85)	−0.29** (0.75)	4.32 (0.92)	−0.30** (0.77)	4.48 (0.86)	−0.27** (0.74)
EQ-5D	0.85 (0.17)	−0.01 (0.15)	0.77 (0.22)	0.01 (0.19)	0.84 (0.17)	−0.02* (0.15)
Primary care costs	555 (827)	−16 (701)	810 (1,153)	−149 (1,191)	565 (751)	97 (1,092)
Outpatient hospital care	326 (662)	−104 (643)	725 (1,342)	−34 (1,728)	269 (492)	76* (657)
Inpatient hospital care^$^	471 (3,009)	−334 (3,120)	1,064 (5,012)	932 (9,807)	476 (3,085)	742 (10,225)
Medication costs	269 (275)	0 (248)	493 (423)	1 (289)	356 (351)	4 (255)
Total healthcare utilization costs	1,600 (3,665)	−447 (3,663)	3052 (5,787)	754 (10,204)	1,653 (3,525)	918 (10,574)
Travelling costs	63 (145)	73 (571)	89 (221)	−48* (185)	72 (226)	−5* (312)
Productivity costs	3,542 (11,480)	−1,685 (10,076)	1,119 (6,401)	−86 (6,964)	1,405 (7,646)	−368 (6,743)
Total costs	3,633 (10,091)	−317 (11,593)	4,421 (10,657)	159 (13,876)	2,911 (8,201)	725 (13,874)

The table presents the mean (SD) and the mean difference (SD) between baseline and follow-up measurements; ^$^inpatient hospital care costs include also emergency care costs; *(p < 0.05); **(p < 0.01); the differences are calculated subtracting the costs at baseline from the costs at follow-up; primary care costs included contacts with GP, nurse practitioner, nurse, dietician, physiotherapist, podiatrist, lifestyle coach, etc.

Table 6 presents the yearly costs and outcomes of patients enrolled in CVR-DMPs that target different populations (i.e. primary prevention, secondary prevention, or both types of prevention). After 12 months, the hospital costs of patients included in DMPs targeting both types of CVR prevention increased by €819 within a year. Further, patients in DMPs for secondary prevention and for both types of prevention had €48 and €5 lower travelling costs, respectively. The travelling costs at baseline in these two types of DMPs were also higher compared to the primary prevention DMPs.

### Determinants of changes in HR-QoL and costs within DMPs

The results from the generalized linear mixed models are presented in Table 7. Model one shows that a greater improvement in EQ-5D utility is significantly predicted by a lower baseline EQ-5D score, a higher baseline physical activity level, a greater increase in physical activity, and a greater increase in self-efficacy. One additional day with more than 30 minutes of physical activity leads to a 3% higher EQ-5D utility and 1 unit of increase in self-efficacy score leads to a 4% higher EQ-5D utility. In contrast, patients with COPD had 7% less improvement in EQ-5D and patients with multi-morbidity 5% less.

**Table 7:** Determinants of changes in HR-QoL and health care utilization costs

	**Model 1**		**Model 2**	
	**Change in EQ-5D**		**Change in health care utilization costs**	
	**e(b)**	**p**	**e(b)**	**p**
Intercept	1.04	0.744	104192.98	<0.001
EQ-5D/	0.60	<0.001		
Costs (in 000’s) baseline			0.95	<0.001
Age	1.00	0.408	1.00	0.130
Physical activity (1–7 highest)	1.02	0.023	1.00	0.777
Change in physical activity	1.03	0.001	1.00	0.639
PACIC (1–5 highest)	0.99	0.474	1.02	0.247
Change in PACIC	1.00	0.830	1.00	0.843
Self-efficacy (1–6 highest)	1.00	0.956	0.98	0.107
Change self-efficacy	1.04	0.032	1.01	0.730
Quit smoking (1 = yes)	1.04	0.119	1.07	0.104
Multi-morbidity (1 = yes)	0.95	0.019	1.06	<0.001
COPD* (1 = yes)	0.93	<0.001	1.01	0.541
DMII* (1 = yes)	0.99	0.576	1.02	0.460
Additional payment (1 = yes)	0.99	0.468	0.99	0.491
N	820		843	
R^2^ patient level	0.36		0.73	
R^2^ DMP level	0.56		0.78	

*the reference category is CVR-DMP; Note: the predictor variables COPD-DMP, DMII-DMP, and Additional payment are on the DMP level. All other variables are on the patient level.

The best predictors of change in health care utilization costs were health care utilization costs at baseline and the presence of multi-morbidity (model 2). If costs were €1000 higher at baseline, the increase was 5% less. In case of multi-morbidity, the cost increase was 6% higher. The variance in the dependent variables explained by models 1 and 2 at the DMP and the patient level was relatively high.

### Comparing costs and effects between DMPs

The results from the cost-utility analysis taking the health care and societal perspective are presented in Table 8. This table shows that the most effective DMP for CVR primary prevention, combined primary and secondary CVR prevention, and DMII led to statistically significant cost savings when compared to the least effective DMP in the same disease category (i.e. more than 95% of bootstrap replications in the southern quadrants). It also shows there is large variation in incremental costs (ranging from €-721 to €1,716) and incremental QALYs (ranging from 0.003 to 0.013) between the best and the worst DMP within a disease category. Due to the very small incremental QALYs the ICERs are very large. The 5000 bootstrapped ICERs plotted on the CE plane showed that there is large uncertainty around the estimated mean ICER. Considering the CVR- primary prevention sample, 97% of the 5,000 simulated ICERs were in the southern half of the CE plane indicating lower incremental costs while the reverse was observed for the COPD sample (79% of the 5,000 bootstrapped ICERS were on the Northern CE plane).

**Table 8:** Results from the cost-utility analysis

	Most effective VS least effective DMP*	**Incremental costs**	**Incremental QALYs**	**Mean ICER**	**% of 5000 simulated ICERs per quadrant in the CE plane**			
					**NW**	**NE**	**SW**	**SE**
Health care perspective								
CVR-primary^#^	7 VS 4	−534	0.003	−178,539	1	3	41	56
		(297)	(0.021)					
CVR-secondary^$^	1 VS 3	−671	0.012	−56,809	6	21	15	58
		(976)	(0.015)					
CVR-both	2 VS 8	−721	0.005	−148,480	2	2	35	61
		(416)	(0.016)					
COPD	1 VS 4	1,716	0.009	185,747	33	46	11	10
		(2,000)	(0.053)					
DMII	1 VS 3	−677	0.013	−50,234	1	3	14	82
		(398)	(0.013)					
Societal perspective								
CVR-primary^#^	7 VS 4	−1,131	0.003	−377,991	5	12	37	46
		(1,334)	(0.021)					
CVR-secondary^$^	1 VS 3	−153	0.012	−12,929	10	36	11	43
		(1,225)	(0.015)					
CVR-both	2 VS 8	−604	0.005	−124,457	6	8	31	55
		(554)	(0.016)					
COPD	1 VS 4	2,054	0.009	−222,314	34	47	11	9
		(2,371)	(0.053)					
DMII	1 VS 3	−1,735	0.013	−128,790	1	2	14	83
		(1,084)	(0.013)					

*most effective is defined based on the highest incremental QALY and the reverse; ^#^primary prevention for CVD; ^$^secondary prevention for CVD; ICER: incremental cost-effectiveness ratio; CE: cost-effective(ness); best is defined as most effective based on QALYs and worse as the least effective based on the same measurement; the numbers correspond to the DMP numbers in Table 4.

From the societal perspective, the cost-utility results are similar to the results from the health care perspective except that for the secondary CVR prevention samples the uncertainty about the incremental costs became even larger.

### Sensitivity analysis

Table 9 shows the results from the CUA performed excluding the development and implementation costs. The most remarkable change in comparison to the main CUA is that 20% (instead of 4%) of the 5,000 bootstrapped ICERs regarding both CVR prevention DMPs were located on the North quadrant of the CE plane. This change is a result from the higher development and implementation costs of the least effective DMP.

**Table 9:** Results from the cost-utility analysis from the health care perspective excluding the development and implementation costs

	Best DMP VS worse DMP*	**Incremental costs**	**Incremental QALYs**	**Mean ICER**	**% of 5000 simulated ICERs per quadrant in the CE plane**			
					**NW**	**NE**	**SW**	**SE**
CVR-primary^#^	7 VS 4	−407	0.003	−136,077	3	7	39	51
		(330)	(0.021)					
CVR-secondary^$^	1 VS 3	−863	0.012	−73,013	4	14	17	65
		(961)	(0.015)					
CVR-both	2 VS 8	−326	0.005	−67,145	10	10	28	52
		(388)	(0.016)					
COPD	1 VS 4	1,574	0.009	170,390	32	45	12	11
		(1,985)	(0.053)					
DMII	1 VS 3	−430	0.013	−31,942	3	11	12	74
		(402)	(0.013)					

*most effective is defined based on the highest incremental QALY and the reverse; ^#^primary prevention for CVD; ^$^secondary prevention for CVD; ICER: incremental cost-effectiveness ratio; CE: cost-effective(ness); best is defined as most effective based on QALYs and worse as the least effective based on the same measurement; the numbers correspond to the DMP numbers in Table 4.

## Discussion

In this study we have investigated the short-term changes in costs and effects after the implementation of 16 DMPs for three different chronic diseases, namely CVR, COPD, and DMII. We have also explored the within DMP predictors of these changes. Finally, a CUA was performed from the health care and societal perspective comparing each DMP to usual care and comparing the most effective and least effective DMP within five disease categories (i.e. CVR-primary prevention, CVR-secondary prevention, CVR-both types of prevention, COPD, DMII).

Our results show a significant improvement in the level of chronic care integration as measured by the PACIC, in the CVR population (0.10). It improved especially in the DMPs that were directed at primary prevention (0.18) or the combination of primary and secondary prevention (0.10) of cardiovascular diseases. This is promising because patients in these programs had the lowest PACIC scores of the three patient groups. For patients who already had a cardiovascular disease it is probably harder to achieve improvements in integrating care because more (para-) medical disciplines and healthcare sectors become involved. An unexpected result was that the PACIC decreased by 0.23 in the DMII-DMPs. This may be due to difficulties to maintain their high starting level of integrated care, which in turn may be caused by the attention that was paid to quality improvements in diabetes care for the last decade. It would be interesting to examine whether our findings would have been similar if another instrument, for example the Assessment of Chronic Illness Care (ACIC), would have been used to measure the level of chronic care integration. However, we did not include the ACIC in our analysis for two reasons. The first is because this paper focuses on intermediate and final outcomes in patients, not in professionals. The second is that although the two instruments are complementary [19], they both measure the level of integrated care and thus, they correlate [20].

Another interesting finding is that DMPs seem to improve the life-style of patients, in all three disease categories. Patients reported a higher level of physical activity, especially those in DMPs for COPD and CVR management. In addition, the percentage of smokers decreased by more than 5 percentage-point in all disease categories; the decrease was 11 percentage-point in COPD. This reduction is considerably higher as the cessation rate achieved by a physician-advice to stop smoking [21] or the impact of the recent ban on smoking in bars and restaurants [22].

Furthermore, our within-DMP analysis showed a reduction in self-efficacy and generic HR-QoL after the implementation of the DMPs. The slight deterioration (about 0.03 EQ-5D units) in HR-QoL may be explained as a time effect rather than a treatment effect because the HR-QoL of chronic care patients generally tends to decrease over time [23]. Similarly, the decrease in self-efficacy may also be related to the decrease of HR-QoL because deterioration in HR-QoL may worsen self-efficacy [24,25]. Another explanation may be that HR-QoL and self-efficacy are both perceived values that are influenced by the information and knowledge a patient has. DMP interventions included educating patients about their disease, learning them to recognize the early signals of disease-worsening, learning them coping skills and stimulating them to improve their lifestyle. As a result, patients may have become more aware of their impaired health status and their reference point may have shifted.

Our study collected the costs of development and implementation of the DMPs in detail and showed that they can be an important driver of total costs. This is in line with the findings of the few previous studies that have incorporated them in their analysis [3,26,27]. The development and implementation costs per patient were largely driven by the personnel costs. Moreover, the 16 DMPs included in our sample were pioneers in experimenting with DMPs. Therefore, the number of enrolled patients was perhaps not as high in the first year of implementation as the capacity would allow. In the long(er) term, we expect that more patients will be enrolled in the DMPs and caregivers will gain experience in managing and maintaining a DMP. That may lower the implementation costs per patient. Therefore, we would expect more favourable ICERs for the DMPs in the longer term. Within the one-year time frame of our study there are as yet few signals of important changes in the costs of healthcare utilization and productivity loss. But the heterogeneity in DMPs is large with all 3 DMII-DMPs showing a numerical reduction of hospital costs and total health care costs.

The regression analysis indicated that an increase in physical activity was predictive of an increase in HR-QoL. Given the observed increase in physical activity in almost all disease categories, we may expect DMPs to improve HR-QoL in the longer term. We also found that an improvement in self-efficacy was predictive of an improvement in HR-QoL. This creates an opportunity for DMPs to develop and implement strategies to improve the self-efficacy of the patients. Furthermore, patients with multiple morbidities seem to benefit less than patients with one disease. This may imply that the current disease-specific DMPs do not address the needs that patients with multi-morbidity have, and therefore, are less effective for this population. The need for patient-tailored care to address the complex needs of patients with multi-morbidity is extensively addressed in the literature [28,29]. A horizontal integration of DMPs to simultaneously target CVR, COPD, and DMII might be appealing for several reasons. The first one is of course the desire to improve the care for these patients. The second reason is that some components of the DMPs are largely similar, irrespective of the disease. For example, smoking cessation support and physical reactivation can be organized similarly, and adjusted to the specific needs of an individual patient. This avoids inefficiencies and double payments. Another reason is that the number of participants in such a multi-disease DMPs will increase, which will lower the implementation and overhead costs per participant.

We also performed a CUA comparing DMPs within a disease area, which is interesting for decision makers once they have decided to implement a DMP. Then the variability in costs and health outcomes is likely to drive the choice of program. When adopting the health care perspective the CUA showed that the majority of the bootstrapped ICERs in all types of CVR prevention and DMII comparison pairs were located on the South-East quadrant of the CE plane. This indicates that the most effective DMPs had lower costs and positive QALY gains compared to the least effective DMPs in these three disease groups. This finding remained also when the societal perspective was adopted. However, the results concerning the primary CVR prevention and COPD were more difficult to interpret because of the uncertainty about the QALY gains (health care and societal perspective).

As our results showed, the cost-effectiveness of DMPs varies considerably, most likely depending on the components of the program, the target population, the success of the implementation and the costs of managing and operating the program. These are all factors that contractors of DMPs should consider in the negotiation phase. We are planning future analysis aiming to identify the factors that drive the cost-effectiveness of a DMP. These findings could contribute to the on-going debate in the Netherlands on whether the current bundled payment system for single-disease DMPs are an intermediate stage towards population-based financing [6]. Population based financing includes a risk-adjusted fixed budget (either per group of patients or region) to cover all health care provided by multiple professionals from different disciplines. Savings compared to a pre-defined benchmark are often shared between payer and provider. A large variation in the cost-effectiveness of DMPs due to the aforementioned factors, jeopardizes the successful implementation of DMPs as means to achieve integration of chronic care. Thus, a population-based financing with larger scope in terms of covered population and provided interventions, economies of scale that lower operating costs, and consensus of all stakeholders that ensures successful implementation may appear attractive to Dutch policy makers. However, the preconditions to introduce a population-based financing are far from being reached [30] and therefore, the implementation of DMPs on more disease areas is still work in progress.

This study contributes to the growing body of international evidence on integrated care in several ways. First, it highlights the necessity to adopt a broad set of outcome measures and include the most important cost items from different perspectives in the evaluation of DMPs. Second, the findings of our study support the previous studies that concluded that DMPs are positively associated with improvements in patient lifestyle and quality of care [20,31,32]. Third, our finding that DMPs have the potential to become cost-effective in the long-term, and the identification of factors that drive that cost-effectiveness, could inform designers of integrated care programs in other European countries. Fourth, the limitation of disease-specific DMPs to address the needs of complex patients could urge collective initiatives on a European level to develop adequate models of integrated care for this population.

Our study is one of very few studies providing insight into health economic aspects of DMPs that includes such a broad range of outcome measures and cost categories. However, we fully acknowledge the limitations of the study design with respect to causality. At the start of this study there were multiple initiatives to provide integrated care across the entire country, stimulated by the introduction of the bundled payment system and other financial incentives. Therefore it was impossible to create a control group at regional level. It was also difficult to identify control groups within the same organization because of the high risk of contamination [33]. This risk is high because the implementation of a DMP requires changes at an organisational level. For example, redesigning the care-delivery process or training nurses in motivational interviewing affects the entire organisation and the entire target population. Therefore, we did not aim to compare the DMPs to usual care but rather compare different DMPs within a disease category. To optimize comparability, we applied inverse probability weighting and corrected for confounders in multivariate analysis. In addition, our results may be object to regression to the mean bias. However, this bias is probably limited because our sample size is relatively large and the diseases included in our analysis are chronic and progressive. These assumptions are supported by a previous study that found minimal evidence of regression to the mean in COPD-DMPs [34].

## Conclusions

This study of the short-term effects of DMPs found that the implementation of DMPs was associated with improvements in integration of care and lifestyle behaviour, such as physical activity and smoking, of patients with CVR, diabetes and COPD. Since an increase in physical activity and an increase in self-efficacy were predictive of an improvement in HR-QoL, DMPs that aim to improve these are more likely to be effective. This study has also shown the wide variation in development and implementation costs between DMPs and pointed at the importance of economies of scale. On this short term we have not found statistically significant cost savings due to DMPs, but it is likely that it takes more time before the improvements in care lead to reductions in complications and hospitalizations.

### Ethics statement

The study protocol was approved by the ethics committee of the Erasmus University Medical Centre of Rotterdam (September 2009). For more details see Lemmens et al. [8].

## Abbreviations

DMP: Disease management program; DMII: Diabetes mellitus type 2; COPD: Chronic obstructive pulmonary disease; CVR: Cardiovascular risk; ICT: Information and communication technology; PACIC: Patient assessment of chronic illness care; SMAS-S: Self-management ability scale-shorter; QALY: Quality adjusted life year; WHO: World health organization; GP: General practitioner; ICER: Incremental cost-effectiveness ration; CE: Cost-effectiveness; HR-QoL: Health related quality of life; D&I: Development and implementation; ZonMw: Nederlandse Organisatie voor Gezondheidsonderzoek en Zorginnovatie (Netherlands Organization for Health Research and Development); FTE: Full-time equivalent; SES: Socio-economic status.

## Competing interests

The authors declare that they have no competing interests. The research was financially supported by the Netherlands Organization for Health Research and Development (ZonMw, project number 300030201).

## Authors’ contributions

AT designed the study for this paper, collected the data, performed the data analysis, and drafted the manuscript; JMC and APN collected the data and commented on previous drafts of the manuscript; MRvM designed the study for this paper, collected the data, coordinated the study, and commented on previous versions of the manuscript; all authors critiqued and approved the final version of the manuscript.

## Supplementary Material

Additional file 1 Interventions per DMP. Click here for file

Additional file 2 Unit cost prices used in the costs analysis. Click here for file
