# Supplementary material for: Changes in costs and effects after the implementation of disease management programs in the Netherlands: variability and determinants
Source: Cost Eff Resour Alloc. 2014 Jul 28;12:17. doi: 10.1186/1478-7547-12-17 (PMC4118650; doi:10.1186/1478-7547-12-17)
Supplement: Additional file 1 — Interventions per DMP. [file 1478-7547-12-17-S1.docx]

Additional File 1. Interventions per DMP

| Intervention \ DMP | CVR-DMP 1 | CVR-DMP 2 | CVR-DMP 3 | CVR-DMP 4 | CVR-DMP 5 | CVR-DMP 6 | CVR-DMP 7 | CVR-DMP 8 | CVR-DMP 9 | COPD-DMP 1 | COPD-DMP 2 | COPD-DMP 3 | COPD-DMP 4 | DMII-DMP 1 | DMII-DMP 2 | DMII-DMP 3 |
| --- | --- | --- | --- | --- | --- | --- | --- | --- | --- | --- | --- | --- | --- | --- | --- | --- |
| **Organizational support** |  |  |  |  |  |  |  |  |  |  |  |  |  |  |  |  |
| Integrated financing |  |  |  |  |  |  |  |  |  |  |  |  |  |  |  | √ |
| Specific policies and subsidies for foreign population |  |  |  |  |  |  | √ |  |  |  |  |  | √ |  |  |  |
| Sustainable financing agreements with health insurers |  |  |  | √ | √ |  |  |  |  |  |  |  |  |  | √ | √ |
| **Community** |  |  |  |  |  |  |  |  |  |  |  |  |  |  |  |  |
| Communication platform between stakeholder about patients |  |  |  |  |  |  |  |  |  |  |  |  |  |  |  |  |
| Health market |  |  |  |  |  |  |  |  |  |  |  |  |  |  |  |  |
| Cooperation with external community partners |  |  |  | √ |  |  |  |  |  |  |  |  |  |  | √ |  |
| Multidisciplinary and transmural collaboration |  |  |  |  |  |  |  |  |  |  |  | √ |  | √ |  |  |
| Role model in the area |  |  |  |  |  |  |  |  |  |  |  |  |  |  |  |  |
| Regional collaboration for spread of the DMP |  |  |  |  |  |  |  |  |  |  |  |  |  |  | √ |  |
| Treatment and care pathways in outpatient and inpatient care |  |  |  | √ |  | √ |  |  |  |  |  | √ |  | √ |  |  |
| Involvement of patient groups and patient panels in care design |  |  |  | √ |  |  |  |  |  |  |  |  | √ | √ | √ |  |
| Regional training course |  |  |  | √ |  |  |  |  |  |  |  |  |  |  | √ | √ |
| Family participation |  |  |  | √ |  |  |  |  |  |  |  |  |  |  |  |  |
| **Self management** |  |  |  |  |  |  |  |  |  |  |  |  |  |  |  |  |
| Promotion of disease specific information | √ | √ |  | √ |  |  | √ | √ |  |  |  | √ | √ |  |  |  |
| Individual care plan | √ |  |  | √ |  |  |  |  |  |  |  | √ | √ |  |  |  |
| Life-style interventions (e.g. physical activity, diet, smoking cessation) |  |  | √ | √ | √ |  |  | √ |  |  |  | √ | √ |  |  | √ |
| Support of self-management (e.g. internet, email or sms, e-consultation) |  |  |  |  | √ |  |  |  |  |  |  |  |  |  |  |  |
| Tele-monitoring |  |  |  |  |  |  |  |  |  |  |  |  |  |  |  |  |
| Personal coaching | √ |  |  | √ |  |  |  | √ |  |  |  |  | √ | √ |  |  |
| Motivational interviewing | √ |  |  | √ |  | √ |  |  |  |  |  | √ | √ | √ |  |  |
| Informational meetings |  |  |  |  |  |  |  |  |  |  |  |  |  |  |  |  |
| Diagnosis and treatment of mental health issues | √ |  |  | √ |  |  |  | √ |  |  |  |  | √ | √ |  |  |
| Mirror interviews |  |  |  |  |  |  |  |  |  |  |  |  |  |  |  |  |
| Group sessions for patients and family |  |  |  |  |  |  |  |  |  |  |  |  |  |  |  |  |
| Cognitive behavioural therapy |  |  |  |  |  |  |  |  |  |  |  |  |  |  |  |  |
| **Decision Support** |  |  |  |  |  |  |  |  |  |  |  |  |  |  |  |  |
| Care standards / Clinical guidelines | √ | √ |  | √ | √ | √ |  | √ |  | √ | √ | √ | √ | √ | √ |  |
| Uniform treatment protocol in outpatient and inpatient care |  |  |  | √ | √ |  |  |  |  |  |  |  |  | √ | √ |  |
| Training and independence of practise assistants |  |  |  | √ | √ |  |  |  |  |  |  |  | √ | √ | √ |  |
| Professional education and training for care providers |  | √ |  | √ | √ |  |  |  |  |  |  |  | √ | √ | √ |  |
| Automatic mesurement of proces/outcome indicators |  | √ |  | √ | √ |  |  |  |  |  |  |  | √ | √ | √ | √ |
| Development and implementation of care protocols for immigrants |  |  |  | √ |  |  |  |  |  |  |  |  |  |  |  |  |
| Audit and feedback | √ |  |  | √ | √ |  |  |  |  |  |  |  | √ | √ |  |  |
| Periodic evaluation of interventions and goal achievement |  |  |  | √ |  |  |  |  |  |  |  |  |  | √ |  |  |
| Structural participation in knowledge exchange/best practices |  |  |  | √ |  |  |  |  |  |  |  |  |  | √ | √ |  |
| Quality of Life questionnaire |  |  |  | √ |  |  |  |  |  |  |  | √ | √ | √ |  |  |
| Qualitative evaluation of health care via focus-groups with patients |  |  |  | √ |  |  |  |  |  |  |  |  | √ |  |  |  |
| Measurement of patient satisfaction |  | √ |  | √ | √ | √ |  |  |  |  |  |  | √ | √ |  | √ |
| **Delivery System Design** |  |  |  |  |  |  |  |  |  |  |  |  |  |  |  |  |
| Delegation of care from specialist to nurse/care practitioner | √ |  |  | √ | √ |  |  |  |  |  |  | √ | √ | √ |  | √ |
| Substitution of inpatient with outpatient care |  |  |  |  |  |  |  |  |  |  |  |  |  | √ | √ |  |
| Systematic follow-up of patients |  | √ |  | √ | √ |  |  |  |  |  |  | √ | √ | √ |  |  |
| One-stop outpatient clinic |  |  |  | √ |  | √ |  |  |  |  |  |  |  |  |  | √ |
| Specific plan for immigrant population |  |  |  | √ |  |  | √ |  |  |  |  |  |  |  |  |  |
| Expansion of chain care to the secondary care setting |  |  |  |  |  |  |  |  |  |  |  |  |  | √ |  |  |
| Joint consultation hours |  |  |  |  |  |  |  |  |  |  |  |  |  |  |  |  |
| Meetings of different disciplines for exchanging knowledge/information |  |  |  | √ |  |  |  |  |  |  |  |  | √ | √ |  | √ |
| Monitoring of high-risk patients |  |  |  | √ |  |  |  |  |  |  |  |  | √ |  |  |  |
| Board of clients |  |  |  | √ |  |  |  |  |  |  |  |  | √ |  |  | √ |
| Periodic discussion sessions between care professionals (and patients) |  |  |  | √ |  |  | √ |  |  |  |  | √ | √ | √ |  | √ |
| Stepped care method |  |  |  | √ |  |  | √ |  |  |  |  |  |  | √ | √ |  |
| **ICT** |  |  |  |  |  |  |  |  |  |  |  |  |  |  |  |  |
| Electronic Patient Records system with Patient Portal |  |  |  |  |  |  |  |  |  |  |  |  |  |  |  |  |
| Hospital or Practice Information System | √ | √ |  | √ | √ | √ | √ | √ | √ | √ | √ | √ | √ | √ | √ | √ |
| Integrated Chain Information System |  |  |  | √ |  |  |  |  |  |  |  |  |  | √ |  |  |
| Use of ICT for Internal and/or regional benchmarking |  |  |  | √ |  |  |  |  |  |  |  |  | √ | √ | √ |  |
| Create a safe environment for data exchange |  | √ |  | √ |  |  |  |  |  |  |  |  | √ | √ |  | √ |
| Systematic registration by every caregiver |  | √ |  | √ | √ |  |  |  |  |  |  |  |  |  | √ | √ |
| Exchange of information between different care disciplines |  |  |  | √ |  |  |  |  |  |  |  |  | √ | √ |  |  |
