# Supplementary material for: Changes in costs and effects after the implementation of disease management programs in the Netherlands: variability and determinants
Source: Cost Eff Resour Alloc. 2014 Jul 28;12:17. doi: 10.1186/1478-7547-12-17 (PMC4118650; doi:10.1186/1478-7547-12-17)
Supplement: Additional file 2 — Unit cost prices used in the costs analysis. [file 1478-7547-12-17-S2.docx]

Additional File 2: Unit cost prices used in the costs analysis.

|  | 2009 | 2010 | 2011 | 2012 |
| --- | --- | --- | --- | --- |
| Inflation rate-  Dutch Statistics (CBS) | 1,2 | 1,3 | 2,3 | 2,5 |
|  |  |  |  |  |
| GP session | 28 | 28 | 29 | 30 |
| GP home visit | 43 | 44 | 45 | 46 |
| GP phone contact | 14 | 14 | 15 | 15 |
| Inpatient day | 457 | 463 | 474 | 485 |
| Day care treatment | 251 | 254 | 260 | 267 |
| Intensive care unit day | 2,183 | 2,211 | 2,262 | 2,319 |
| Outpatient visit | 72 | 73 | 75 | 76 |
| Emergency room visit | 151 | 153 | 156 | 160 |
| Physical therapy | 36 | 36 | 37 | 38 |
| Speech therapy (session) | 33 | 33 | 34 | 35 |
| Occupational therapy (hour) | 22 | 22 | 23 | 23 |
| Dietary advice (hour) | 27 | 27 | 28 | 29 |
| Home care (hour) | 35 | 35 | 36 | 37 |
| cost/km | 0.20 | 0.20 | 0.21 | 0.21 |
